# Deep Learning-Based Non-Intrusive Commercial Load Monitoring

**DOI:** 10.3390/s22145250

**Published:** 2022-07-13

**Authors:** Mengran Zhou, Shuai Shao, Xu Wang, Ziwei Zhu, Feng Hu

**Affiliations:** School of Electrical and Information Engineering, Anhui University of Science and Technology, Huainan 232001, China; mrzhou@aust.edu.cn (M.Z.); wangxu@aust.edu.cn (X.W.); zwzhu0039@163.com (Z.Z.); hufeng0106@163.com (F.H.)

**Keywords:** non-intrusive load monitoring, commercial load, deep learning, multi-label classification, correlation, imbalance

## Abstract

Commercial load is an essential demand-side resource. Monitoring commercial loads helps not only commercial customers understand their energy usage to improve energy efficiency but also helps electric utilities develop demand-side management strategies to ensure stable operation of the power system. However, existing non-intrusive methods cannot monitor multiple commercial loads simultaneously and do not consider the high correlation and severe imbalance among commercial loads. Therefore, this paper proposes a deep learning-based non-intrusive commercial load monitoring method to solve these problems. The method takes the total power signal of the commercial building as input and directly determines the state and power consumption of several specific appliances. The key elements of the method are a new neural network structure called TTRNet and a new loss function called MLFL. TTRNet is a multi-label classification model that can autonomously learn correlation information through its unique network structure. MLFL is a loss function specifically designed for multi-label classification tasks, which solves the imbalance problem and improves the monitoring accuracy for challenging loads. To validate the proposed method, experiments are performed separately in seen and unseen scenarios using a public dataset. In the seen scenario, the method achieves an average F1 score of 0.957, which is 7.77% better than existing multi-label classification methods. In the unseen scenario, the average F1 score is 0.904, which is 1.92% better than existing methods. The experimental results show that the method proposed in this paper is both effective and practical.

## 1. Introduction

As the energy crisis worsens, more and more renewable energy resources are being connected to the power system [1]. Due to the intermittency and uncertainty of renewable energy resources such as wind and solar energy, this will pose significant challenges to the stable operation of the power system [2]. The primary current solution is demand response, which regulates electricity load by providing appropriate incentives in response to the supply of electricity from renewable energy sources [3]. Commercial load is regarded as an essential demand-side resource due to its flexibility and controllability [4]. How to reasonably and efficiently monitor the commercial load has become the focus of researchers [5]. In the past, commercial users would install electricity meters on electrical equipment in order to know their own electricity consumption information. However, with the advancement of technology, there are more and more electrical equipment in commercial buildings, and the traditional intrusive load monitoring method of installing measuring equipment on each piece of equipment is no longer applicable. To this end, researchers have proposed non-intrusive load monitoring (NILM) [6]. This technology can obtain the power consumption information of the equipment by using the total power consumption information. Compared with intrusive load monitoring, it has the advantages of low cost and easy implementation, and is more cost-effective for commercial users [7]. Therefore, proposing an effective method to realize non-intrusive commercial load monitoring is crucial.

Existing NILM methods are mainly used for residential loads [7]. Commercial loads differ from residential loads in terms of energy consumption and load characteristics, which is the main reason why existing NILM solutions are not directly applicable to commercial loads [8]. The earliest NILM study specifically for commercial loads was proposed by Norford et al. [9]. They do this by matching steady-state and transient changes to known patterns. Their work sparked interest in high-power loads such as heating, ventilation and air conditioning (HVAC) in commercial buildings. Subsequently, Ji et al. [10] proposed a NILM method based on a Fourier series model to determine the hourly end-use of HVAC in commercial buildings. In recent years, there have been attempts to use machine learning algorithms for NILM of commercial loads. Ling et al. [11] and Xiao et al. [12] simultaneously proposed an approach based on random forest, one for disaggregating out the energy consumption of building subsystems and one for disaggregating out the cooling load of buildings. In addition, generative models are also applied to NILM, such as EnerGAN [13] and EnerGAN++ [14]. Henriet et al. [15] were the first to apply generative models to non-intrusive commercial load monitoring. The input signal can also be processed using a graph-based method [16]. While paying attention to models or algorithms, the practicality of the NILM method has also attracted more and more attention. In practical applications, NILM is usually performed at a lower sampling frequency [17]. Rafsanjani et al. [18] uses density-based spatial clustering of applications with noise and quadratic discriminant analysis to perform non-intrusive commercial load monitoring at the occupant level rather than on specific HVAC equipment or systems. Modern commercial buildings generally have building automation systems (BAS), so Zaeri et al. [19] proposed a disaggregation method for the end-use of commercial buildings based on BAS data and multiple linear regression models.

To sum up, traditional classification and regression algorithms still dominate non-intrusive commercial load monitoring. These methods typically require manual feature extraction using domain expert knowledge and are less transferable [20]. Additionally, non-intrusive commercial load monitoring has the following problems: 1. Multiple electrical devices cannot be identified at the same time. Demand response often requires acquiring multiple loads simultaneously. However, due to the large number and variety of devices used by commercial customers, the approach of training a separate model for each load is no longer applicable [21]. 2. The potential correlation between electrical equipment cannot be considered. For example, a high correlation between commercial air conditioning units may lead to simultaneous startup or shutdown events, violating the one-at-a-time assumption [8]. 3. The unbalanced phenomenon of the state of electrical equipment cannot be considered. Under different power consumption scenarios, different types of devices have different startup or shutdown times. For example, some devices are turned off for a long time and only turned on for a short time, which affects the monitoring accuracy [21].

In response to the above challenges, this paper proposes a deep learning method for non-intrusive commercial load monitoring, which can directly obtain the operating status and power consumption of multiple internal electrical devices from the overall power consumption of commercial buildings without a complex features project. The method includes a novel deep learning framework called Transformer-Temporal Pooling-RethinkNet (TTRNet) and a novel loss function called Multi-Label Focal Loss (MLFL). Considering the need for commercial loads to participate in demand response and the power consumption logic of commercial customers, two different NILM evaluation scenarios, called “seen” and “unseen”, were created and tested to demonstrate that the proposed method can achieve high performance with good transferability in terms of equipment state identification and energy decomposition. The following are the main contributions of this work:

1. The proposed deep learning method for non-intrusive commercial load monitoring is based on a multi-label classification task, which can simultaneously identify the operating status of multiple commercial electrical devices and decompose the power consumption of multiple devices, reducing the time cost of existing commercial NILM methods. Moreover, the label correlation and class imbalance problems are solved from the model framework and training method, respectively, to improve monitoring accuracy.
2. Compared with the existing models, the encoder part of TTRNet designs the structure of stacking multiple identical blocks, and each block is composed of a transformer encoder and max-pooling to automatically extract the characteristics of the total input power sequence. A Temporal Pooling block is added between the encoder and decoder to provide more detailed features by adding contextual information when identifying the activation state. Finally, RethinkNet is introduced in the decoder part to enhance the learning of interrelationships between each power-consuming device and improve the accuracy of multi-label classification.
3. By improving the existing single-label Focal Loss for multi-label classification tasks, not only the load imbalance problem in NILM is solved, but also the accuracy of hard-to-identify loads is improved.

The rest of this paper is organized as follows: Section 2 presents and compares the related work. Section 3 describes the proposed deep learning method in detail. Section 4 describes the experimental steps in the public dataset. Section 5 presents the experimental results, and Section 6 presents the analysis. Section 7 concludes and proposes some possible further work.

## 2. Related Work

Recurrent Neural Networks (RNN) and their variants have been the dominant method for solving problems with sequence data. However, the structure of RNN leads to weaknesses in parallel computing. In addition, one of the biggest drawbacks of RNN is the problem of vanishing gradients and exploding gradients when the sequence is too long. To overcome these limitations, the attention mechanism is introduced as a solution [22]. On this basis, Vaswani et al. [23] proposed a new simple network architecture, namely a Transformer. Transformers currently have success in field such as natural language processing, computer vision, and time series forecasting, sparking great interest in the NILM community. Lin et al. [24] were the first to apply a Transformer to NILM and simultaneously proposed two different networks, one containing only multiple encoder blocks and the other keeping the original encoder-decoder structure. Experiments demonstrate that a Transformer improves NILM accuracy, robustness, and training cost compared to existing RNNs and Convolutional Neural Networks (CNN). Yue et al. [25] proposed an improved objective function specially designed for NILM learning and a Transformer representation architecture based on bidirectional encoders and also achieved better results than the existing methods. In addition to using off-the-shelf Transformers, the architecture and training methods can be improved to better fit the NILM task. Yue et al. [26] further replaced the original self-attention with local attention to improve the poor performance of capturing local signal patterns, while Sykiotis et al. [27] proposed the use of unsupervised pre-training and downstream task fine-tuning to improve prediction accuracy and reduce training time, both with better results. Several NILM methods mentioned above are compared in Table 1. The comparison shows that existing Transformer-based methods either use encoders or improve them. These methods have only been tested for residential loads and have not been evaluated for commercial loads. Furthermore, these methods are mainly used for regression or single-label classification tasks rather than multi-label classification tasks. Therefore, this paper pioneers the use of Transformers for non-intrusive commercial load monitoring and multi-label classification tasks.

In recent years, multi-label classification methods have become increasingly popular in NILM. Instead of obtaining the power consumption of individual electrical devices for training purposes, the multi-label classification-based NILM approach requires only the operational status of individual devices, making the load monitoring process truly non-intrusive. In addition, device status can be easily obtained from automated smart building records or from commercial building managers. Moreover, this method can output multiple loads simultaneously, which can reduce the time cost and facilitate the practical application of NILM disaggregated data. Massidda et al. [28] proposed Temporal Pooling NILM (TP-NILM), a CNN-based network architecture for multi-label classification tasks, which uses the Temporal Pooling module to collect contextual information, which is an adaptation and simplification of the pyramidal scene parsing network for image semantic segmentation proposed by Zhao et al. [29]. In addition to this, there are some ways to use context awareness for NILM [30]. Nolasco et al. [31] proposed DeepDFML-NILM, a complete NILM deep learning method that includes a high-frequency signal detection part and a feature extraction part, in addition to the most important multi-label classification part. Verma et al. [32] proposed a multi-label LSTM autoencoder (LSTM-AE) for NILM. All the above multi-label classification methods are directly implemented by CNN or RNN without considering the correlation of labels and the imbalance of categories, i.e., correlation and imbalance between loads in NILM tasks. This approach may be feasible for residential loads but not for commercial loads with strong correlations and severe state imbalances, so it is considered in this paper.

Label correlation and category imbalance are two major problems in multi-label classification research. Usually, one expects to improve the correct rate of multi-label classification by learning the correlation between labels or solving the category imbalance. Yang et al. [33] envisioned to model the correlation between labels by treating the multi-label classification task as a sequence generation problem. Yang et al. [34] proposed a new cost-sensitive multi-label classification (CSMLC) algorithm, called RethinkNet, by mimicking the human rethinking process. They use the structure of RNN as the main part of the model. In addressing imbalances, Lin et al. [35] proposed a new loss function Focal Loss and applied it to dense object detection. In the field of NILM, Zhou et al. [36] proposed a method that considers both label relevance and class imbalance. The method is implemented by a CNN-LSTM-RF model, where the class imbalance problem is mainly solved by a compound reweighting approach, while the label relevance problem is solved by the method proposed by [33]. However, the model was tested only in the seen scenario of residential load, and thus the generalization ability of the model could not be tested. In contrast, this paper conducted experiments on unseen scenarios.

## 3. Methodology

**Figure 1:**
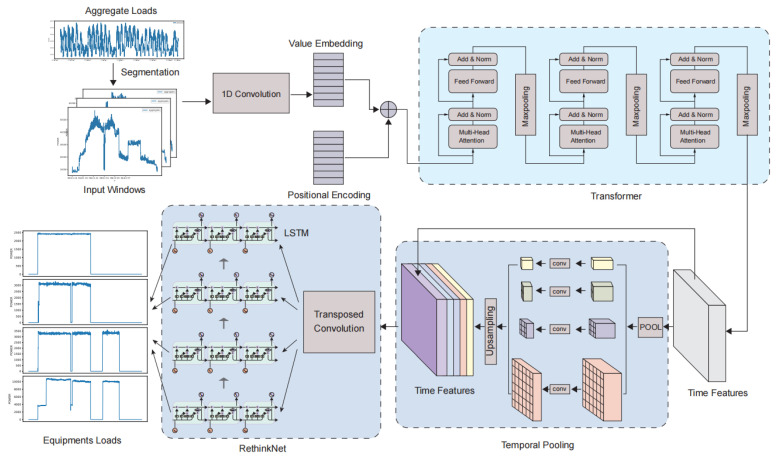
The TTRNet model architecture.

The method proposed in this paper has two main parts: a neural network architecture, TTRNet, and a loss function, MLFL. Where TTRNet consists of four components: Input Embedding, Transformer, Temporal Pooling, and RethinkNet, the overall structure is shown in Figure 1. Each part is described separately below.

### 3.1. Input Embedding

First, perform input embedding on the aggregated loads. Because the Transformer cannot learn the order of the input information, the loading sequence cannot be passed directly to the Transformer part. Input embedding consists of two parts: value embedding and positional encoding.

The value embedding transforms the input sequence into a 256-dimensional vector using a 1D convolution with a kernel width of 3 and a stride of 1.

(1) $$E_{v}\left(x\right)=Conv\left(x\right)$$

The positional encoding is used to generate the relative and absolute position information for the input sequence.

(2) $$\begin{array}{c} E_{p}\left(p,2i\right)=\sin\left(\frac{p}{10000^{2i/d}}\right)\\ E_{p}\left(p,2i+1\right)=\cos\left(\frac{p}{10000^{2i/d}}\right) \end{array}$$

Finally, the feature vector of the value embedding transformation is added to the position vector generated by the positional encoding to add position information to the input sequence.

(3) $$E_{i}\left(x\right)=\alpha E_{v}\left(x\right)+E_{p}\left(p,\right)$$

where α is a factor that balances the value embedding and the positional encoding. The input sequence will be normalized during data preprocessing later, so here α=1.

### 3.2. Transformer

**Figure 2:**
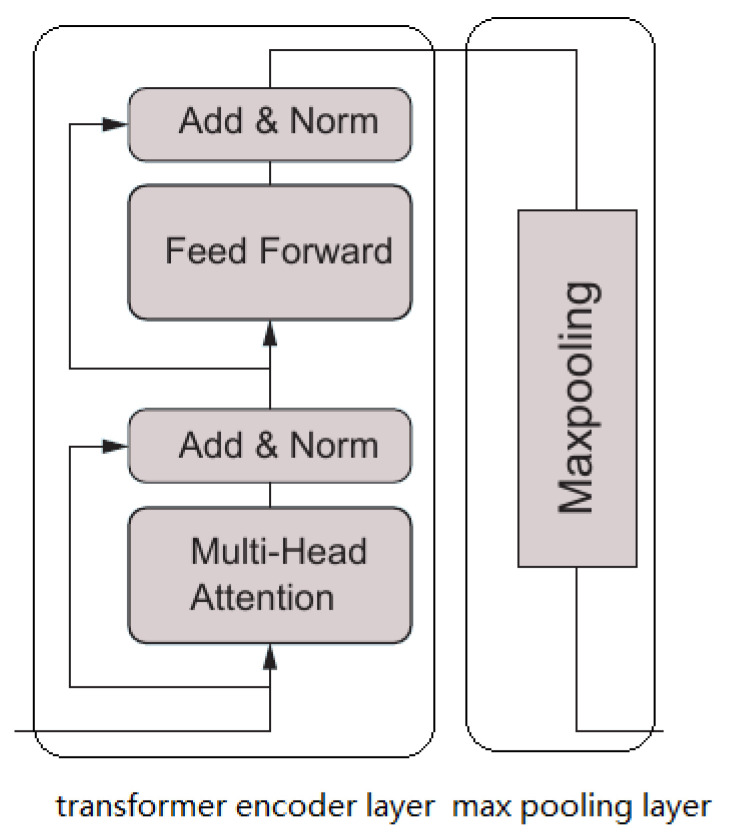
A Transformer block of the TTRNet.

The Transformer part of TTRNet, i.e., the encoder part of TTRNet, is used to extract input features. This part uses the Transformer’s encoder structure. It consists of a stack of N identical modified encoder blocks, each of which includes a Transformer encoder layer and a max-pooling layer, whose structure is shown in Figure 2.

Each Transformer encoder layer consists of two sublayers. The first layer is multi-head attention, and the second layer is a feedforward network. Each sublayer performs skip connections followed by layer normalization. Multi-head attention consists of several parallel self-attentions. Self-attention operates on the input Q, K, and V matrices. First, Q and K are multiplied, then divided by the square root of the hidden size; softmax is used to generate soft attention and multiplied by V to get the final weighted value matrix.

(4) $$\mathrm{Attention}\left(Q,K,V\right)=\mathrm{softmax}\left(\frac{QK^{T}}{\sqrt{d_{k}}}\right)V$$

(5) $$\begin{array}{c} MultiHead\left(Q,K,V\right)=Concat\left(head_{1},\ldots ,head_{h}\right)W^{O}\\ \;\;\;\;\;\;\;\mathrm{where}\;{\mathrm{head}}_{i}=Attention\left(QW_{i}^{Q},KW_{i}^{K},VW_{i}^{V}\right) \end{array}$$

where each multi-head attention uses h = 8 self-attention heads.

The effect of each max-pooling layer is to halve the temporal resolution of the output of the previous Transformer encoder layer. In this study, 3 Transformer blocks were used, so the temporal resolution of the input sequence was eventually reduced to one-eighth of its original value.

### 3.3. Temporal Pooling

**Figure 3:**
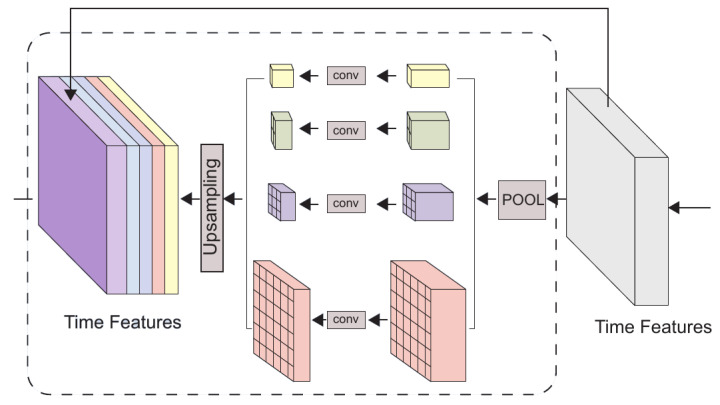
The Temporal Pooling module.

TTRNet adds a Temporal Pooling between the traditional encoder-decoder structure for introducing more contextual information, which is shown in Figure 3.

As shown in the figure above, the Temporal Pooling module has five channels, one of which does nothing and keeps the original features unchanged. The remaining four channels perform Temporal Pooling operations. In these four channels, feature vectors are first passed through average pooling layers with kernel sizes of 5, 10, 20, and 30 to reduce the temporal resolution. Then, each channel performs a 1D convolution to reduce the feature dimension to a quarter of the original dimension. The Temporal Pooling operation for each channel can be expressed as:

(6) TP(x)=BN(ReLU(Conv(pool(x))))

At the end of each of the four channels, linear upsampling is performed so that the output of the Temporal Pooling block has the same precise time resolution as the encoder output. Finally, the five channels are concatenated to form a new feature vector. The total number of features with contextual information is doubled compared to the number of features fed into the Temporal Pooling module.

### 3.4. RethinkNet

**Figure 4:**
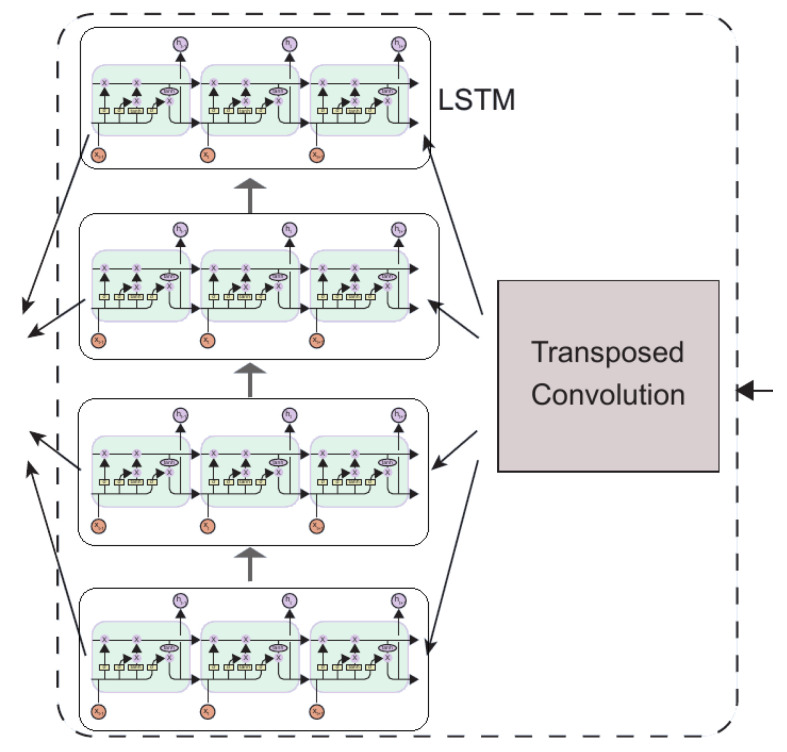
The RethinkNet module.

The main role of the RethinkNet module is to decode the previously extracted feature vector into an output sequence with multiple labels. Figure 4 illustrates the design scheme of this part, which consists mainly of a transposed convolution, multiple recurrent network layers, and finally a fully connected layer.

The output of the Temporal Pooling module is the input to the transposed convolution, which uses a kernel size and stride of 8 to increase the temporal resolution of features while reducing the number of features. After layer normalization, these features are fed into multiple recurrent networks. Multiple recurrent network layers are arranged in parallel, the input of each layer is from the transposed convolution, and the hidden state of each layer is from the previous recurrent network layer.

RethinkNet is a memory label correlation model across recursive network layers. RethinkNet forms initial guesses in the first recursive grid layer stores them in memory and iteratively corrects them using label correlation. Each layer of the recursive network is an iterative process that “rethinking” through multiple iterations.

(7) $${\hat{y}}^{\left(t\right)}=\sigma \left(W_{1}x+W_{2}{\hat{y}}^{\left(t-1\right)}\right)$$

where $W_{1}x$ is the feature term, which comes from transposed convolution, and $W_{2}{\hat{y}}^{\left(t-1\right)}$ is the memory term that converts the previous predictions into the current label vector space. The label correlation information is stored in global memory, so the order of the labels does not affect classification results.

In this study, a long short-term memory network (LSTM) was selected as the recurrent network layer. This is because LSTMs can guarantee good results when dealing with long sequences of data no matter how many iterations are made. In this study, four LSTM layers were used. The output of LSTM is linearly transformed by the fully connected layer, and finally activated by the sigmoid function to realize multi-label classification.

### 3.5. MLFL

In practical applications, existing methods often perform poorly in practice due to the different proportions of different operating states of different electrical equipment of commercial customers. This can be solved by changing the loss function. Larger weights are assigned to smaller proportions of samples, and smaller weights are assigned to larger proportions of samples. Increasing the proportion of small-scale samples in the overall loss function guides network training to favor small-scale samples, thereby improving the classification accuracy of small-scale samples.

In addition to this, in the later stages of training, the most identifiable loads are correctly classified, while only a few challenging loads are misclassified. Likewise, the classification accuracy of hard-to-identify loads can be improved by weighting.

Focal Loss is an excellent solution to this problem, but the original Focal Loss was applied to single-label classification tasks. In this study, Focal Loss is improved by designing weights for each label separately, making it usable for multi-label classification tasks. The improved MLFL is shown below:

(8) $$\begin{array}{cc} L_{MLFL}=-\frac{1}{N}\sum_{n=1}^{N} & \sum_{l=1}^{L}\alpha_{l}*y_{nl}*{\left(1-p_{nl}\right)}^{\gamma }*\log\left(p_{nl}\right)+\left(1-y_{nl}\right)*p_{nl}^{\gamma }*\log\left(1-p_{nl}\right) \end{array}$$

where $\alpha_{l}$ represents the proportion of negative to positive samples for the *l*th label. γ is the weight in the loss function for challenging loads that can take on values between 0 and 5. In this work, γ is set to 2.

## 4. Experiment

This section focuses on the experiments performed to verify the effectiveness of the proposed method. Section 4.1 describes the dataset used. Section 4.2 describes the data preprocessing process. Section 4.3 details the experiments conducted in this paper for two different evaluation scenarios (seen and unseen). Section 4.4 presents evaluation metrics describing the experimental results.

### 4.1. Dataset

**Figure 5:**
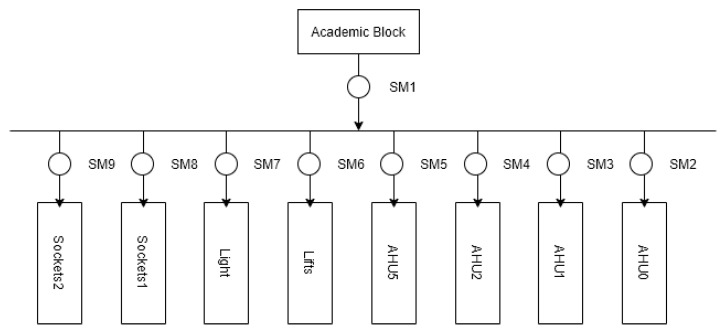
Academic Block’s smart meter installation location instructions.

In this study, the above methods are experimented with using the Commercial Building Energy Dataset (COMBED) [8]. This dataset includes real-world data collected from smart meters deployed in different buildings and subsystems of IIITD. These real-world figures include total electricity consumption per building, high energy loads, and total electricity consumption per floor. The Academic Block can be thought of as a commercial building similar to an IT office. Like other commercial buildings, it mainly includes air conditioning load, lighting load, elevator load, etc. Since the air conditioning load is often used for demand response, this paper takes the air handling unit (AHU) of the Academic Block as the research object. AHU0, AHU1, AHU2, and AHU5 are four loads in different power consumption scenarios. The smart meter installation location of the Academic Block is shown in Figure 5, and the collected data information is shown in Table 2.

In the field of NILM, it is common to use not only real data but also synthetic data in order to improve the generalization performance of the model [37]. Here, data such as lighting and elevator loads collected from sub-meters are used as noise. The main meter data for the new commercial building is formed by subtracting this noise from the actual main meter data. This synthetic data retains the original characteristics of the AHU load in it, with some noise and other factors only found in real data, so it can be experimented with as a regular commercial building. Compared to training with real data only, a mixture of real and synthetic data can improve the transferability of the above methods to applications in different types of commercial buildings. The ratio of synthetic data to generated real data is 4:1, and the total measurement properties of the resulting dataset are shown in Table 3.

### 4.2. Data Preprocessing

Data for each commercial air conditioning unit and each commercial building in the dataset were preprocessed before the experiments were performed. The first step filters measurement errors by removing outliers from the active power measurement data for each air conditioning unit, limiting the maximum power to the values provided in Table 4. The second step obtains the activation status of each air conditioning unit. In general, a device can be considered operating when the power it absorbs exceeds a certain threshold λ [37]. However, due to the complexity of the commercial air conditioning equipment, the absorbed power may drop below the threshold for short periods of time without actually shutting down the equipment. Therefore, we specify a minimum shutdown time of $\mu_{0}$. During this time frame, a commercial air conditioning unit is considered to be truly off if its power remains below a threshold. Finally, to filter false activations caused by metering issues, we specify a minimum on-time $\mu_{1}$. During this period, if the power of the commercial air conditioner is above the threshold, it is considered to be truly on. Table 4 shows the activation thresholds λ for the above commercial air conditioning equipment, as well as the values for the minimum on time $\mu_{0}$ and the minimum time $\mu_{1}$. In the third step, the master meter data for each building is divided into multiple input time windows of size 480, i.e., 4 h, which can contain at least one activation state change. Finally, in the fourth step, the main meter data for each building is normalized by dividing the total load by the maximum power value of 60,000 W.

### 4.3. Training and Testing

This study was conducted under the following two different NILM assessment scenarios. (1) Seen. The composition of the commercial load is known; that is, trained on multiple loads in multiple commercial buildings, tested on multiple loads in one of the commercial buildings; (2) Unseen. The composition of the commercial load is unknown; that is, trained on multiple loads in multiple commercial buildings and tested on multiple loads in another commercial building which is not trained. This scenario is more in line with practical applications.

Therefore, each commercial building in the dataset described earlier was divided into three consecutive time periods containing 70%, 15%, and 15% of the measurements; 70% is used for training, and the remaining 30% is used for validation and testing. It is important to note that the tests in the unseen scenario were performed on the 100% value of the building. The specific division of the dataset in this study is shown in Table 5.

In the seen scenario, we train the first part of building 1 using real data and the first part of buildings 2, 3, 4, and 5 using synthetic data. We verify the second part of Building 1. During the validation phase, the network parameters are saved when a new minimum value is reached throughout the training period. Finally, we test the third part of Building 1. Training and testing the model in this context enables the evaluation of the model’s ability to identify and decompose the load when the composition of the commercial load is known.

In the unseen scenario, we train the first part of buildings 1 and 5, validate the second part of buildings 1 and 5, and also save the network weights when the loss function reaches a new minimum. Building 2 is used to simulate an unknown building and test all of its data. Training and testing the model, in this case, enables the evaluation of the generalization performance of the model. Since the original purpose of NILM research is to apply to unknown buildings in real life, the results in this scenario have greater application value.

All experiments are performed on a Linux host with the following specifications, CPU: 15-core AMD EPYC 7543 32-core processor 30 GB; Graphics: RTX A5000*1 24 GB. In these two different evaluation scenarios, the network parameters are optimized using the Adam optimization method, which uses the gradient descent technique with a learning rate of 10–5 and a batch size of 32. The above hyperparameter combinations do not reflect the maximum accuracy of the test case, as the purpose of this experiment is mainly to verify the effectiveness of the proposed technique.

### 4.4. Evaluation Metrics

In this paper, five event detection (ED) metrics and two energy estimation (EE) metrics are used to comprehensively evaluate the performance of the NILM method.

The ED indicator measures the performance of the algorithm in detecting device activation. True Positives (TP) are the number of moments when AHU is correctly identified as active. True Negative (TN) is the number of moments when the AHU is correctly assessed to be closed. False Positives (FP) represent the number of moments when the AHU was not working but was reported as working. False Negatives (FN) are the number of moments when the AHU actually worked but was incorrectly evaluated as off. Precision (9) represents the ratio of TP to the number of all moments evaluated as active. Recall (10) represents the ratio of TP to the actual activation moment. Accuracy (11) represents the ratio of all correctly evaluated moments to the total number of moments. F1 Score (12) combines precision and recall, with a maximum value of 1 and a minimum value of 0. The closer to 1, the better the recognition of the load state. The Mathews Correlation Coefficient (MCC) (13) represents the performance of the classification method in the range [−1, 1], where 1 means accurate classification, 0 means random classification and −1 means misclassification.

(9) $$\mathrm{Precision}\;=\frac{TP}{TP+FP}$$

(10) $$\mathrm{Recall}\;=\frac{TP}{TP+FN}$$

(11) $$\mathrm{Accuracy}\;=\frac{TP+TN}{TP+TN+FP+FN}$$

(12) $$F_{1}=2\frac{\;\mathrm{Precision}\;\times \;\mathrm{Recall}\;}{\;\mathrm{Precision}\;+\;\mathrm{Recall}\;}$$

(13) $$\mathrm{MCC}\;=\frac{\;TP\;\times TN-FP\times FN}{\sqrt{\left(TP+FP)(TP+FN)(TN+FP)(TN+FN\right)}}$$

The EE metric estimates the precise amount of energy consumed by the device. This paper uses mean absolute error (MAE) (14) and signal aggregation error (SAE) (15) to measure the accuracy of active power estimates for individual devices. MAE measures the average deviation of estimated power relative to actual power at each instant. At the same time, SAE measures the relative error of the power estimates used throughout the evaluation period.

(14) $$MAE\;=\frac{1}{N}\sum |{\hat{y}}_{}\left(t_{}\right)-y_{}\left(t_{}\right)|$$

(15) $$SAE\;=\frac{\sum {\hat{y}}_{}\left(t_{}\right)-\sum y_{}\left(t_{}\right)}{\sum y_{}\left(t_{}\right)}$$

where $y_{}\left(t_{}\right)$ denotes the true value of the power and ${\hat{y}}_{}\left(t_{}\right)$ denotes the estimated value of the power.

## 5. Results

This section describes and explains the experimental results for two different NILM evaluation scenarios, the seen and unseen scenarios, respectively.

### 5.1. Seen

A total of twenty experiments were performed, and the results were averaged. The 90% interval for multiple results is also reported to show the stability of the method. Table 6 shows the performance of the method for identifying states, estimating instantaneous power consumption and total power consumption in the scenarios seen. As can be seen from the table, all five ED indicators except individual were above 0.9, indicating that the method effectively identified the activation states of the four AHUs. The average F1 score is 0.957, the average precision is 0.988, the average recall is 0.931, the average precision is 0.976, and the average MCC is 0.943, all with good stability. For the estimated instantaneous power consumption, the average MAE of the four AHUs is 197.13. Among them, AHU5 has the worst result because AHU5 has the highest operating power. For the estimated total power consumption, the average SAE of the four AHUs is −0.049.

**Figure 6:**
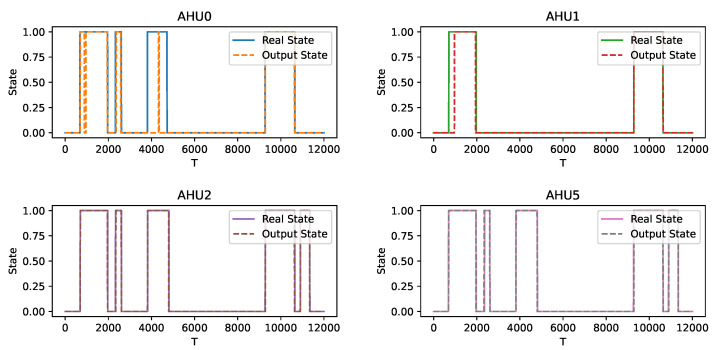
Comparison of the output state and the real state in the seen scenario.

Then, the output states are compared with the real states, respectively. Figure 6 shows the identification of the states of the four AHUs in this scenario. It can be seen that there are errors in the identification of several activations of AHU0 and AHU1, while the identification of both AHU0 and AHU1 is perfect, which may be due to the large differences in the operation of AHU0 and AHU1. Overall, the method is effective in capturing the changes in the operating state of each AHU.

### 5.2. Unseen

Twenty experiments were also conducted in the unseen scenario. Table 7 shows the method’s performance in identifying the state and estimating the instantaneous and total power consumption in this scenario. The average F1 score is 0.904, the average accuracy is 0.932, the average recall is 0.878, the average accuracy is 0.919, and the average MCC is 0.834. For estimating the transient power consumption, the average MAE of the four AHUs is 433.24. For estimating the total power consumption, the average SAE of the four AHUs is −0.058. With a guaranteed average F1 score of 0.904, the accuracy of AHU2 and AHU5 remain at 0.940 and 0.983, respectively, indicating that the method is still effective in identifying unseen scenarios.

**Figure 7:**
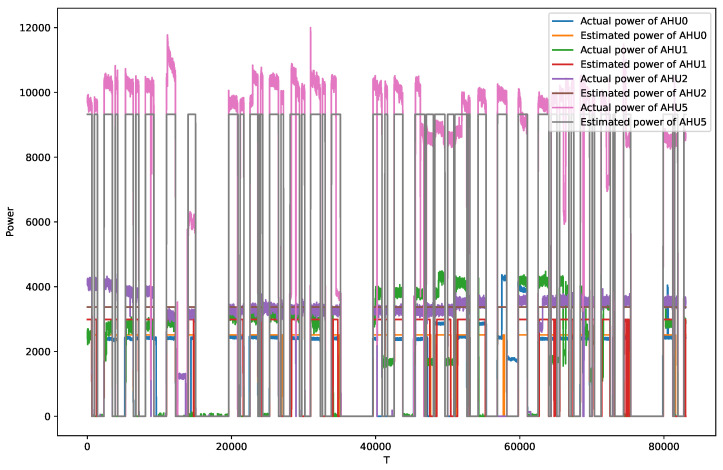
Comparison of the instantaneous power estimates and the actual power in the unseen scenario.

As can be seen from the above table, the method performs relatively poorly in estimating the instantaneous power in the unseen scenario. Figure 7 shows the power estimates of the method for the four AHUs. The poor MAE was obtained because these AHUs had high operating power. However, the total power estimate for the whole monitoring period is still excellent, as evidenced by the average SAE of −0.058.

## 6. Discussion

This study aims to enable non-intrusive monitoring of multiple commercial loads simultaneously. According to the research results, in the see scene: the average score of F1 is 0.957, the average score of SAE is −0.049, and in the unseen scene: the average score of F1 is 0.904, and the average score of SAE is −0.058. The above results show that the method proposed in this paper has achieved good results in the state identification and power decomposition of all four commercial loads, indicating that the method can achieve the preliminary purpose of the experiment.

Furthermore, the basic idea of this study is to improve monitoring accuracy by considering the correlation and imbalance of commercial loads. In this approach, correlations are solved by RethinkNet and imbalances are solved by MLFL. To this end, extensive ablation studies were performed to verify the role of each component, and the results are shown in Table 8.

The TP-NILM [28] was used as the baseline. The accuracy of AHU2 is particularly poor compared with the other three loads, indicating that AHU2 is a load that is difficult to identify. Model A removes the Temporal Pooling module of the baseline and becomes a structure composed entirely of deep CNN. The result is a significant drop in F1 scores for all four loads, proving that the contextual information does have the effect of improving the results. Model B uses a Transformer as the encoder and trade-offs each load to improve the average F1 score from 0.844 to 0.890 in the baseline. This result proves that a Transformer can extract load features better than a CNN in NILM. Model C adds RethinkNet to the baseline and improves the average result, indicating the importance of considering label relevance in multi-label classification. Part of the load accuracy decreases, which may be related to the correlation difference. Model D was trained using MLFL on top of Model C and continued to improve the average F1 score, indicating that solving the load imbalance problem can effectively improve the classification accuracy. Especially for AHU2, it has increased from 0.772 to 0.897, indicating that MLFL can improve the monitoring performance of challenging loads. Model E adds RethinkNet to Model B, and the increase in average F1 score proves that Transformer and RethinkNet work better together. Finally, the complete TTRNet model is obtained using MLFL based on model E. The average F1 score is improved from 0.888 in the baseline to 0.957, which is an improvement of 7.77%. After adding MLFL, the results of AHU1 will be slightly reduced, which can also be seen in the comparison of model C and model D. This may be affected by the current MLFL weight parameter combination. The current setting is enough to prove the effectiveness of this method, and it will be optimized by adjusting parameters in the future.

This method is also thoroughly compared with existing NILM methods based on multi-label classification. Here, the existing methods are chosen from CNN and TP-NILM, i.e., Model A and the baseline mentioned before. Table 9 and Table 10 indicate the performance comparison of different methods in the seen and unseen scenarios, respectively.

By comparing the seven performance metrics with existing methods, most of the metrics of TTRNet are far better than other methods, proving that the comprehensive performance of the proposed method in this paper is the best. However, the present method still has some shortcomings. This method is slightly less effective in identifying AHU1, and the specific reasons have been explained above. The most significant advantage of this method is its good power estimation performance while ensuring the correct identification of the load state, especially for high power loads. The MAE of AHU5 in the seen scenario is 380.09, which is 123.08% better than the 847.91 of TP-NILM, and the MAE of AHU5 in the unseen scenario is 592.14, which is 781.18% better than the 1055.06 of TP-NILM. The evaluation of the unseen scenario is more meaningful because it is consistent with the application of NILM in real life. In this scenario, the average F1 score of TTRNet is 0.904, which is 1.92% better than the 0.887 of TP-NILM. Although the results show that the improvement of the proposed method over existing methods is not particularly large, the main reason is that the data of the used public dataset are relatively ideal. In this case, the advantages of this method are still reflected, and the effect of this method will be more obvious when it is applied to more realistic data in the future. To sum up, the method proposed in this paper has higher practical value than existing methods.

## 7. Conclusions and Future Work

From the perspective of demand response, this paper introduces the research value of NILM for commercial loads. Then, this paper summarizes the algorithms used and the problems of existing non-invasive commercial load monitoring methods. It is found that the field is currently stuck in traditional algorithm research. These traditional algorithms cannot meet the requirements of today’s demand response, which requires the simultaneous acquisition of multiple loads. Further, the correlation and imbalance of commercial loads are the main issues that limit the application of non-intrusive methods. Therefore, this paper proposes a deep learning approach to address several such problems simultaneously.

The main idea of the approach is to propose a deep learning network, TTRNet, whose inputs aggregate commercial load information and end-to-end directly outputs the subcommercial load sequences contained therein. The design of TTRNet is divided into three main steps. First, a deformer-based encoder is designed to enhance the extraction of time series features by invoking and improving the latest techniques in NLP. Secondly, a Temporal Pooling module is designed to pool features extracted from the encoder at different time scales, thus increasing the perceptual domain without affecting the temporal resolution of the output. Finally, a RethinkNet module is designed to utilize its unique rethinking process to learn relevance while achieving the final multi-label classification. The original Focal Loss is improved to adapt it to multi-label classification tasks and solve the load imbalance problem and improve the accuracy of challenging loads.

In this study, the method was tested using the public dataset COMBED. To simulate a realistic NILM application environment, a seen scenario and an unseen scenario were designed separately. Then, several experiments were conducted under these two different evaluation scenarios. Not only ED metrics but also EE metrics were used to evaluate the performance of the method comprehensively. The experimental results show that the method has high performance and stability in identifying the activation state, estimating the instantaneous power and total power in both seen and unseen scenarios. In addition, ablation experiments were conducted in this study to demonstrate the role of each component. The experimental results show that considering the correlation and imbalance of loads can effectively improve the accuracy of the NILM method for non-invasive commercial loads. This not only provides a theoretical basis for the NILM study of commercial loads but also provides new ideas for the NILM study of other types of loads.

In future work, two approaches will be taken: First, the study will be extended to multivariate heterogeneous commercial loads. This study can be seen as a study of multivariate homogeneous loads. More factors need to be considered to realize NILM for multivariate heterogeneous commercial loads based on this study. Second, the research approach will focus on lightweight models to speed up training and inference and investigate more efficient optimization processes to improve the quality of monitoring for challenging loads.

## Figures and Tables

**Table 1:** Comparison of Transformer-based NILM methods.

Reference	Name	Publication Date	Dataset	Framework		
				Task	Main Components	Loss
Lin et al. [24]	MA-net and MAED-net	August 2020	REDD	r and c	Encoders and Decoders	MSE
Yue et al. [25]	BERT4NILM	November 2020	REDD and UK-DALE	r and c	Encoders	MSE +KL divergence +soft-margin
Yue et al. [26]	ELTransformer	March 2022	REDD and UK-DALE	r and c	Encoders	MSE
Sykiotis et al. [27]	ELECTRIcity	April 2022	REDD and UK-DALE and Refit	r and c	Encoders	MSE +KL divergence

**Table 2:** Attribute information for electrical load data collected from the Academic Block.

Dataset Properties	Value
Number of main meters	1
Number of sub-meters	8
Sampling frequency	30 s
Sampling range	1 June 2014–1 July 2014

**Table 3:** The main meter data information of commercial buildings in the synthetic dataset.

	Actual Location	Main Meter Load Composition	Main Meter Measurement Composition
Building1	Academic Block	Total Load	SM1
Building2	Academic Block	Total load-Lifts load	SM1-SM6
Building3	Academic Block	Total load-Light load	SM1-SM7
Building4	Academic Block	Total load-Socket1 load	SM1-SM8
Building5	Academic Block	Total load-Socket2 load	SM1-SM9

**Table 4:** Parameter information for obtaining the activation state of the AHU device.

	AHU0	AHU1	AHU2	AHU5
Max. power limit (W)	5000	4500	4500	12,000
Active power threshold λ (W)	500	450	450	1200
Min. OFF duration $\mu_{0}$ (min)	15	15	15	15
Min. ON duration $\mu_{1}$ (min)	15	15	15	15

**Table 5:** Dataset partitioning for the seen and unseen scenarios.

		Building 1	Building 2	Building 3	Building 4	Building 5
seen	Training (%)	70	70	70	70	70
	Validation (%)	15	-	-	-	-
	Testing (%)	15	-	-	-	-
unseen	Training (%)	70	-	-	-	70
	Validation (%)	15	-	-	-	15
	Testing (%)	-	100	-	-	-

**Table 6:** Performance of the method in the seen scenario, including the average and 90% interval of each load over twenty experiments and the average of all loads.

	AHU0		AHU1		AHU2		AHU5		Avg
	Avg	90% Interval	Avg	90% Interval	Avg	90% Interval	Avg	90% Interval	
F1	0.892	(0.817,0.944)	0.951	(0.865,0.996)	0.992	(0.991,0.993)	0.991	(0.983,0.994)	0.957
Precision	0.986	(0.964,1.000)	0.978	(0.884,1.000)	0.998	(0.997,0.999)	0.990	(0.973,0.995)	0.988
Recall	0.819	(0.691,0.912)	0.928	(0.836,0.996)	0.986	(0.983,0.989)	0.992	(0.987,0.994)	0.931
Accuracy	0.938	(0.901,0.965)	0.979	(0.948,0.998)	0.994	(0.993,0.995)	0.994	(0.988,0.996)	0.976
MCC	0.859	(0.777,0.920)	0.940	(0.845,0.995)	0.988	(0.985,0.990)	0.986	(0.974,0.991)	0.943
MAE	185.19	(121.69,270.67)	142.76	(108.08,220.30)	80.41	(77.92,83.34)	380.09	(365.31,438.53)	197.13
SAE	−0.148	(−0.292,−0.037)	−0.059	(−0.174,0.071)	−0.057	(−0.061,−0.053)	0.070	(0.060,0.090)	−0.049

**Table 7:** Performance of the method in the unseen scenario, including the average and 90% interval of each load over twenty experiments and the average of all loads.

	AHU0		AHU0		AHU0		AHU0		Avg
	Avg	90% Interval	Avg	90% Interval	Avg	90% Interval	Avg	90% Interval	
F1	0.864	(0.854,0.874)	0.827	(0.812,0.843)	0.940	(0.938,0.942)	0.983	(0.982,0.985)	0.904
Precision	0.910	(0.877,0.926)	0.871	(0.829,0.916)	0.955	(0.951,0.960)	0.989	(0.983,0.995)	0.932
Recall	0.823	(0.791,0.862)	0.788	(0.743,0.829)	0.926	(0.917,0.931)	0.978	(0.971,0.985)	0.878
Accuracy	0.883	(0.877,0.889)	0.867	(0.857,0.880)	0.941	(0.940,0.943)	0.984	(0.982,0.985)	0.919
MCC	0.765	(0.754,0.776)	0.722	(0.701,0.749)	0.883	(0.880,0.887)	0.967	(0.964,0.971)	0.834
MAE	359.17	(345.36,371.95)	515.57	(470.77,551.56)	266.09	(260.46,271.35)	592.14	(577.86,617.02)	433.24
SAE	−0.095	(−0.148,−0.015)	−0.094	(−0.169,0.001)	−0.030	(−0.045,−0.021)	−0.012	(−0.024,−0.001)	−0.058

**Table 8:** Ablation studies of different components of TTRNet in the seen scenarios.

Model	Transformer	Temporal Pooling	RethinkNet	Focal Loss	AHU0	AHU1	AHU2	AHU5	Avg
Baseline	−	√	−	−	0.926	0.772	0.923	0.930	0.888
ModelA	−	−	−	−	0.871	0.683	0.871	0.880	0.826
ModelB	√	√	−	−	0.898	0.840	0.901	0.922	0.890
ModelC	−	√	√	−	0.943	0.831	0.899	0.890	0.891
ModelD	−	√	√	√	0.904	0.897	0.921	0.931	0.913
ModelE	√	√	√	−	0.924	0.844	0.964	0.977	0.927
TTRNet	√	√	√	√	0.892	0.957	0.992	0.991	0.957

**Table 9:** Performance comparison of different multi-label classification NILM methods in seen scenarios. The number in bold is the largest of the three model comparisons.

Device	Model	F1score	Precision	Recall	Accuracy	MCC	MAE	SAE
AHU0	CNN	0.871	0.783	0.982	0.907	0.812	273.71	0.286
	TP-NILM	**0.926**	0.875	**0.985**	**0.948**	**0.891**	**169.85**	0.160
	**TTRNet**	0.892	**0.986**	0.819	0.938	0.859	185.19	**−0.148**
AHU1	CNN	0.683	0.529	**0.971**	0.800	0.612	702.30	0.836
	TP-NILM	0.772	0.650	0.959	0.872	0.716	484.95	0.485
	**TTRNet**	**0.951**	**0.978**	0.928	**0.979**	**0.940**	**142.76**	**−0.059**
AHU2	CNN	0.871	0.781	0.985	0.894	0.798	422.14	0.206
	TP-NILM	0.923	0.873	0.984	0.938	0.879	272.84	0.088
	**TTRNet**	**0.992**	**0.998**	**0.986**	**0.994**	**0.988**	**80.41**	**−0.057**
AHU5	CNN	0.880	0.795	0.985	0.902	0.813	1235.75	0.325
	TP-NILM	0.930	0.874	**0.996**	0.945	0.891	847.91	0.223
	**TTRNet**	**0.991**	**0.990**	0.992	**0.994**	**0.986**	**380.09**	**0.070**

**Table 10:** Performance comparison of different multi-label classification NILM methods in unseen scenarios. The number in bold is the largest of the three model comparisons.

Device	Model	F1score	Precision	Recall	Accuracy	MCC	MAE	SAE
AHU0	CNN	0.862	0.834	0.894	0.871	0.744	393.80	0.072
	TP-NILM	**0.890**	0.873	**0.910**	**0.899**	**0.798**	**325.52**	**0.043**
	**TTRNet**	0.864	**0.910**	0.823	0.883	0.765	359.17	−0.095
AHU1	CNN	0.763	0.735	0.793	0.801	0.593	692.26	0.080
	TP-NILM	0.822	0.831	**0.814**	0.858	0.704	537.67	**−0.019**
	**TTRNet**	**0.827**	**0.871**	0.788	**0.867**	**0.722**	**515.57**	−0.094
AHU2	CNN	0.862	0.835	0.891	0.858	0.718	551.67	0.067
	TP-NILM	0.903	0.927	0.881	0.906	0.813	384.15	−0.048
	**TTRNet**	**0.940**	**0.955**	**0.926**	**0.941**	**0.883**	**266.09**	**−0.030**
AHU5	CNN	0.901	0.882	0.921	0.900	0.801	1371.48	0.044
	TP-NILM	0.933	0.935	0.933	0.934	0.869	1055.06	**−0.002**
	**TTRNet**	**0.983**	**0.989**	**0.978**	**0.984**	**0.967**	**592.14**	−0.012

## Data Availability

Not applicable.
